# TWEAK-binding autoantibodies are generated during psoriatic arthritis and are not influenced by anti-TNF therapy

**DOI:** 10.1186/s12967-016-0923-8

**Published:** 2016-06-23

**Authors:** Sandrine Guis, Philippe Berbis, Delphine Stephan, Daniel Bertin, Florent Amatore, Nathalie Balandraud, Nathalie Lesavre, Sophie Desplat-Jégo

**Affiliations:** Department of Rheumatology 1, CHU Sainte Marguerite, 270, Bld de Sainte Marguerite, 13009 Marseille, France; CRMBM-CEMEREM UMR 7339, MSK Group, Faculté de Médecine de la Timone, Aix-Marseille Université, 27 Bld Jean Moulin, 13385 Marseille, France; Department of Dermatology, Hopital Nord, Aix-Marseille Université, Marseille, France; NICN, CNRS, UMR7259, Aix-Marseille Université, Marseille, France; Centre d’Investigation Clinique 1409, AP-HM, Aix-Marseille Université, Marseille, France; Service d’Immunologie, Pôle de Biologie, Hôpital de la Conception, Assistance Publique – Hôpitaux de Marseille, Marseille, France

**Keywords:** TWEAK, Psoriatic arthritis, Autoantibodies, Anti-cytokine, Anti-TNF therapy

## Abstract

**Background:**

TNF weakly inducer of apoptosis (TWEAK) is member of the TNF ligand superfamily. Various data support that TWEAK produced by synovial macrophages may contribute to synovitis observed in psoriatic arthritis (PsoA). In PsoA, anti-TNF therapy has been successful in agreement with the key role of TNF in the pathogenesis and the generation by PsoA patients of anti-TNF autoantibodies referred as “beneficial autoimmunity to pro-inflammatory mediators”. However, the role of TNF-alpha in the regulation of TWEAK modulation of inflammation during PsoA remains unknown.

**Methods:**

We have studied level course during anti-TNF therapy of serum soluble TWEAK. In the same cohort, we have investigated the generation of TWEAK-binding autoantibodies by PsoA patients before and after anti-TNF therapy.

**Results:**

Patients with PsoA had significantly higher serum levels of TWEAK compared with controls [respective means (±SEM) were 645 pg/ml (64) and 467 pg/ml (23); (p = 0.006)] but serum soluble TWEAK levels were not correlated with BASDAI (Spearman’s coefficients <0.003, p > 0.05). Our study showed that soluble TWEAK levels were not modulated by etanercept therapy [respective Means (±SEM) were 605 (95) (week 12) and 744 (97) (week 24) pg/ml; (p > 0.23)]. Anti-TWEAK autoantibodies were detected in 9/13 (69.2 %) PsoA patients at inclusion and only in 3/57 (5.3 %) healthy blood donors (p < 0.0001). These circulating antibodies were persistent in PsoA patients and detected at similar levels during etanercept therapy. Moreover we showed that they had a down regulating effect on CCL-2 secretion by endothelial cells stimulated by rh TWEAK in vitro.

**Conclusion:**

Our study revealed that during psoriatic arthritis (1) serum TWEAK was up regulated and (2) TWEAK-binding autoantibodies are generated. Both parameters were not influenced by anti-TNF therapy and persisted at high levels during anti-TNF therapy. For the first time we described here TWEAK-binding IgG autoantibodies with a down regulating effect on CCL-2 secretion by endothelial cells stimulated by rh TWEAK in vitro. Finally, our results suggest that TWEAK may be involved in PsoA pathogeny.

*Trial registration* This clinical trial was approved by the local Ethics Committee “Comité de Protection des Personnes Sud-Méditerranée V” with the registration number: 2011-002954-29, and French health minister registration number AFSSAPS A110784-42 obtained the 08/22/2011. This clinical trial is registered in Clinical trial.gov under the number: NCT02164214

## Background

TNF weakly inducer of apoptosis (TWEAK) is a type II-transmembrane protein, member of the TNF ligand superfamily that can be cleaved to function as a soluble cytokine [1]. Depending on target cell type, TWEAK triggers multiple cellular responses ranging from modulation of inflammation to cell death when it binds to its main receptor, Fn14. Our team has been the first to describe pro-inflammatory effects of TWEAK during central nervous system inflammation and to show protective effect of TWEAK/Fn14 blockade in a multiple sclerosis murine model [2–4]. Similarly, inhibition of TWEAK has been shown to reduce systemic inflammation in other models of autoimmune/chronic inflammatory diseases such as rheumatoid arthritis (RA) or systemic lupus erythematosus [5, 6]. TWEAK is lowly expressed in steady state conditions but during both acute and chronic inflammation TWEAK expression is highly upregulated in many tissues and peritoneal macrophages [3, 7–9]. Various data support the possibility that TWEAK produced by synovial macrophages may contribute to chronic synovitis in animal models and in humans [10–12]. TWEAK may be an actor of inflammatory arthritis pathogenesis by promoting inflammation and angiogenesis. Psoriatic arthritis (PsoA) is a complex and multifaceted chronic inflammatory disease that involves synovial tissue, entheses, skin and nails. This frequent inflammatory arthritis is known to adopt a variable clinical course that may range from mild symptoms requiring minimal intervention to a disabling course leading to loss of function for which treatment strategies targeting inflammation are recommended [13–15]. In this context, anti-TNF therapy has been successful in PsoA concordant with the key pro-inflammatory role of TNF in the pathogenesis of this disease and the generation by PsoA patients of neutralizing anti-TNF autoantibodies has been referred as “beneficial autoimmunity to pro-inflammatory mediators”. In 2010, Van Kuijk et al. have described a high expression of TWEAK and Fn14 in the inflammatory synovial of PsoA and RA patients and have noted the persistence of this expression after anti-TNF therapy [12]. However a direct role of TNF-alpha inhibition in the regulation of TWEAK expression or biological effects remains unknown. Beneficial effects of anti-TNF therapy in inflammatory arthritis could be mediated in part by blocking TNF produced by synovial macrophages but also in part by modulating TWEAK which is also produced by these cells. To assess if anti-TNF therapy affects TWEAK modulation of inflammation during PsoA, we have studied level course during anti-TNF therapy of serum soluble TWEAK. In the same cohort, we have investigated the generation of TWEAK-binding autoantibodies by PsoA patients before and after anti-TNF therapy.

## Patients and methods

### Patients

Twenty-one patients with PsoA who attended the Division of Dermatology and Rheumatology of the Aix-Marseille University Hospital were involved in this prospective study. For inclusion the patients have been examined by both a dermatologist and a rheumatologist. The PsoA was diagnosed according to the CASPAR (ClASsification of Psoriatic ARthritis) group criteria [15]. All the included patients fulfilled the later criteria and were eligible at time of inclusion for anti-TNF therapy [16]. Then, all the patients received a dose of 50 mg once a week of etanercept by subcutaneous injection. Blood samples were collected by venous puncture for each patients at the inclusion time and at 12th and 24th weeks of etanercept therapy. Among these 21 patients, thirteen (7 women and 6 men) have received the complete clinical and biological follow-up as determined in our research protocol. After blood centrifugation, serum samples have been collected, aliquoted and rapidly stored at −80 °C until analysis. Control blood samples were obtained from 57 healthy blood donors with no evidence of autoimmune/chronic inflammatory disease (38 women and 19 men). At the time of blood sample collection for PsoA patients, the erythrocyte sedimentation rate (ESR) and serum C reactive protein concentration were evaluated and the disease activity was assessed according to the BASDAI [17]. Responders were defined as patients with a BASDAI at 12th decreased at least of 50 % or two points in comparison with BASDAI at the beginning of the treatment. Informed consent was obtained from all patients. The procedures followed were approved by the local Ethics Committee (ref CPP 2011-002954-29, ref AFSSAPS A110784-42, Clinical trial.gov number: NCT02164214).

### Quantification of soluble TWEAK and CCL-2 by ELISA

Serum concentrations of soluble TWEAK were determined using commercial ELISA kit purchased from Bender Medsystems, Vienna, Austria, according to the manufacturer’s protocol. The limit of detection of the test was 16 pg/ml. Culture supernatant concentrations of soluble CCL-2 were determined using commercial ELISA kit purchased from Peprotech (Neuilly sur Seine, France) according to the manufacturer’s instructions. The limit of detection of the test was 8 pg/ml. The absorbance was read using a spectrophotometer (Infinite™ TECAN, Mannedorf, Switzerland) at a wavelength of respectively 450 and 405 nm.

### Cell culture and reagents

Human Embryonic Kidney 293 cells (HEK293) and Human umbilical vein endothelial cells (HUVECs) were obtained from ATCC (Molsheim, France) and were respectively cultivated in DMEM with 10 % FCS and 1 % penicillin–streptomycin (Invitrogen, Illkirch, France) and EBM-2 basal medium supplemented with EGM-Plus bullet kit (Lonza, Basel, Switzerland). Recombinant human TWEAK was purchased from Peprotech, Neuilly-sur-Seine, France.

### Detection of TWEAK-binding autoantibodies

#### Western blot

Recombinant human TWEAK (300 ng) was blotted during a 15 % sodium dodecyl sulphate polyacrylamide gel electrophoresis using a Mini-protean system (Bio-Rad, Hercules, CA, USA) and transferred onto nitrocellulose Hybond-C membrane (Amersham Biosciences, Buckinghamshire, UK). The nitrocellulose membrane was then cut into 0.5 cm-wide strips. After blocking, strips were incubated with 1:50 diluted patient serum at 4 °C overnight. After washing, membranes were incubated with a biotin-conjugated anti-human IgG antibody (Jackson Immunoresearch, West Grove, PA), and then with alkaline phosphatase conjugated streptavidin (Jackson Immunoresearch, West Grove, PA). Finally, the strips were incubated with a revelation solution containing nitro-blue tetrazolium and 5-bromo-4-chloro-3′-indolyphosphate (both from Euromedex, Souffelweyersheim, France). In case of positivity at the screening dilution, serum of patients have been further diluted up to 1:1000.

#### Indirect Immunofluorescence

HEK 293 cells were plated in 6-well plates in DMEM Glutamax, 10 % FCS, 1 % penicillin/streptomycin (ThermoFisher scientific, Waltham, MA, USA). The cells were transfected with Jet Pei transfection reagent (Ozyme, Saint-Quentin en Yvelines, France) and pJST773-45-fl-hu-TWEAK plasmid coding for TWEAK, a kind gift from Linda Burkly, (Biogen Idec, Cambridge, MA, USA) which contains full length human TWEAK amino acid 1-248 in the mammalian expression vector CH269. The full length human TWEAK coding sequence was generated by PCR using full length TWEAK cDNA as template and primers containing Not1 sites. The PCR fragment was cloned into the Not1 site of CH269 in the proper orientation and the DNA sequence confirmed. The parental expression vector is a derivative of pCEP4 (Invitrogen). As such, the plasmid pJST773 expresses TWEAK under the control of the CMV promoter and SV40 polyadenylation sequence and is capable of episomal replication due to the presence of the EBV oriP. The plasmid is ampicillin resistant in *E. coli*. Twenty-four hours after transfection, cells were plated on 8-wells microscope slides (ThermoFisher scientific, Waltham, MA, USA). At confluence, the medium was replaced by OptiMEM (ThermoFisher scientific, Waltham, MA, USA) during 12 h and then cells were fixed by PFA 4 %. After blocking, slides were incubated with 1:50 diluted patient serum in PBS BSA 3 % at room temperature. After washing, slides were incubated with FITC-conjugated anti human IgG (Biorad, Hercules, CA, USA) for 1 h at room temperature. Nuclei were stained with Hoechst 33342 nucleic acid stain (ThermoFisher scientific, Waltham, MA, USA). Sections were mounted using Prolong Gold anti fading reagent (ThermoFisher scientific, Waltham, MA, USA). Images were acquired and processed using a light-emitting diode (LED) fluorescence microscope DM1000 (Leica, Wetzlar, Germany) equipped with a digital camera (Fraen, Reading, MA, USA).

### Affinity purification of TWEAK-binding IgG

IgGs from serum of one TWEAK-binding antibody positive PsoA patient were purified on Affi-Gel Protein A MAPS II columns (Bio-Rad, CA, USA) in accordance with the instructions of the manufacturer. This patient had high titers of TWEAK-binding antibody as detected by immunoblotting experiments and the serum used for purification was obtained at the time of inclusion before etanercept treatment. IgG eluted fraction was concentrated with Amicon Ultra 15 mL (Millipore, Molsheim, France) before being applied to the TWEAK affinity column made from Pierce NHS activated Agarose resin columns (ThermoScientific (Massachusetts, UE) and rh TWEAK. Purified total serum IgG were incubated during 2 h at room temperature on the column and then the TWEAK column was washed with PBS (0,1 M phosphate sodium, 0,15 M NaCl pH 7,2) before bound anti-TWEAK IgG were eluted by using 0.1 M glycine–HCl buffer (pH 2.5) and were directly neutralized with 1 M Tris (pH 9). TWEAK-binding IgG were concentrated with Amicon Ultra 4 mL (Millipore, Molsheim, France) and IgG concentration of the fraction was quantified by immunonephelometry on a Siemens Healthcare analyzer (BN Prospec, Siemens Healthcare, Saint Denis, France). The specificity of the eluted material was further confirmed by its ability to bind rh TWEAK in immunoblotting experiment.

### Evaluation of TWEAK-binding IgG effect on CCL-2 secretion by endothelial cells

Human Umbilical Vein Endothelial Cells were incubated for 24 h with rh TWEAK (100 ng/ml) or not (medium condition) in a serum-free culture medium. During this exposure, in other wells, cells were also co-incubated with a commercially available goat polyclonal blocking anti-TWEAK antibody (R&D systems, Minneapolis, MN, USA) (5 µg/ml) or with purified human serum IgG (Sigma Aldrich (Saint Louis, MO, USA) (50 ng/ml) (negative for TWEAK-binding IgG homemade test by western blotting) (both control conditions) or with purified PsoA patient TWEAK-binding IgG (50 ng/ml). Supernatants were collected, centrifuged and stored at −80 °C until quantification of CCL-2 by ELISA. All samples were analyzed in duplicate.

### Statistical analysis

Sensitivity was measured as the probability of a positive result in a patient with PsoA. Specificity was measured as the probability of a negative result in a non-PsoA patient. All statistical analyses were performed using GraphPad Prism^®^ Program Version 6.05 (GraphPad Software Inc., San Diego, CA, USA). Results of soluble TWEAK or CCL-2 (mean ± standard error of the mean) represent duplicate measurements. Groups were compared using the Mann–Whitney nonparametric U-test or independent *t* test as appropriate. Spearman’s rank correlation coefficient was used to test the correlation between serum TWEAK or anti-TWEAK antibodies and other parameters. The p values less than 0.05 were considered statistically significant.

### Ethics section

This clinical trial was approved by the local Ethics Committee “Comité de Protection des Personnes Sud-Méditerranée V” with the registration number: 2011-002954-29, and French minister registration number AFSSAPS A110784-42 obtained the 08/22/2011. This clinical trial is registered in Clinical trial.gov under the number: NCT02164214.

## Results

### Characteristics of the cohort

Table 1 shows the patient characteristics of the PsoA patient cohort. Men and women have been included in equal numbers. Serum of 57 healthy blood donors were also analyzed. The mean age of these control subjects (19 men/38 women) was 41.4 years (range from 27 to 55 years). Most PsoA patients had longstanding disease since 9 patients out of 13 had a disease duration ≥2 years. Ten patients displayed comparable low ESR while the 3 other one had ESR ≥15 mm/h. The CRP levels were found above 5 mg/l for 6 patients. The BASDAI at baseline ranged from 3 to 8 and 10 out of 13 patients were responders to etanercept as indicated by the decrease of their BASDAI at 12 weeks of etanercept administration.

**Table 1:** Characteristics of patients with PsoA

Characteristics	PsoA (n = 13)
Age (years) (range)	50.9 (40–66)
Men/women	6/7
Disease duration (years) (range)	6 (1–22)
BASDAI at baseline (range)	5.8 (3–8)
ESR (mm/h) at baseline (range)	14.5 (1–49)
CRP (mg/l) at baseline (range)	15.1 (1–60)
BASDAI at 12th week (range)	3.7 (0–7.1)
BASDAI at 24th week (range)	3.4 (0.4–8.2)
Responders/non responders	10/3

### Increased levels of serum TWEAK during PsoA before and after anti-TNF treatment

**Fig. 1:**
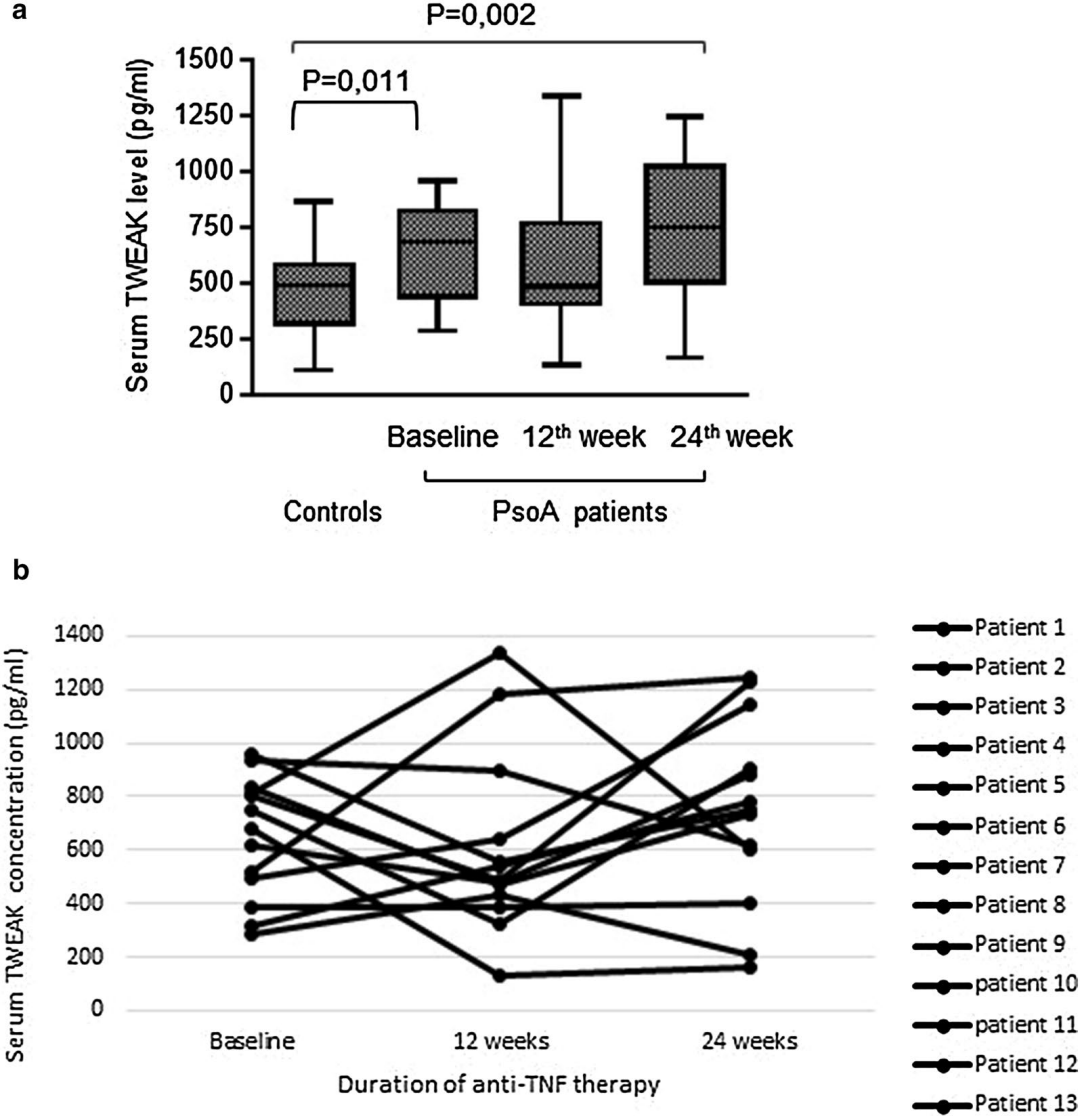
Serum TWEAK levels in patients with PsoA (n = 13) and controls (n = 57). Serum samples have been collected at baseline and 12 and 24 weeks after the initiation of etanercept therapy. TWEAK levels have been evaluated with a commercially available ELISA kit. **a** Median is indicated for each group as a *black line* in the box. **b** Evolution over time of TWEAK levels is represented for each patient

Serum TWEAK levels were significantly higher in PsoA patients at baseline (645 ± 64 pg/ml) and after 24 weeks of etanercept administration (744 ± 97 pg/ml) than in healthy controls (467 ± 23 pg/ml; p < 0.05) (Fig. 1a). During anti-TNF therapy, serum TWEAK levels are not statistically modified in comparison with baseline levels (645 ± 64 pg/ml) neither at 12th week (605 ± 95 pg/ml; p = 0.44) nor at 24th week (744 ± 97 pg/ml; p = 0.55) (Fig. 1a). The evolution profile of serum TWEAK levels during anti-TNF therapy differs for each patient to others as represented in Fig. 1b.

### No evidence for relationship of serum TWEAK levels to response to anti-TNF treatment

**Fig. 2:**
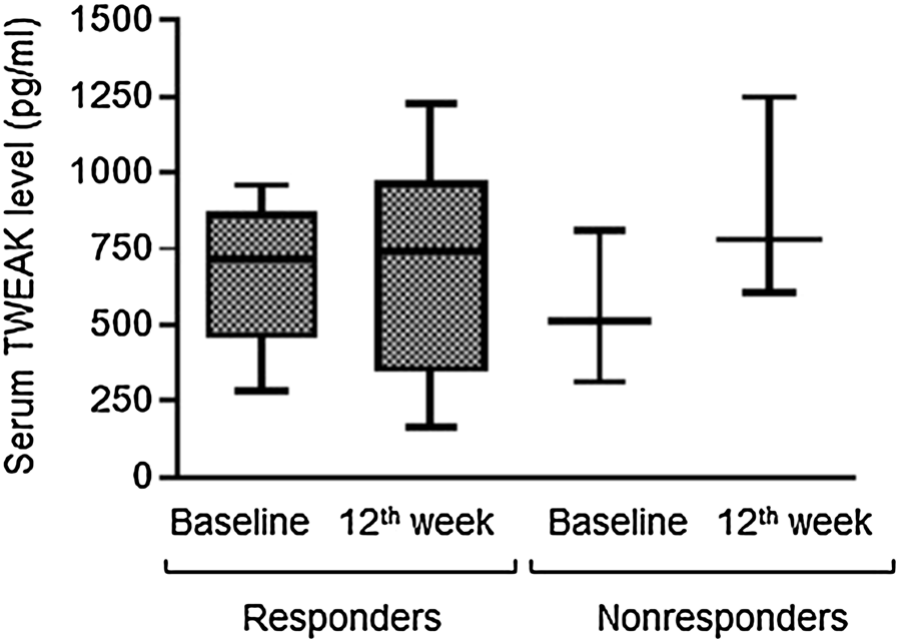
Relationship of serum TWEAK level to response to anti-TNF treatment. Responders (n = 10) are defined as patients with a minimal decrease of BASDAI of 50 % or 2 points at 12th week of treatment. *Black lines* in the *box* indicate the median of values

All the PsoA patients of our study received etanercept treatment after the initial blood sampling and 10 of these (76.9 %) achieved responder criteria at the 12th week of etanercept treatment. When patients were divided into responders and nonresponders, the sample size of the nonresponder group was too low for applying statistical tests but we have observed that there was no obvious difference neither in baseline serum TWEAK levels (responders: 675 ± 72 pg/ml vs. nonresponders: 547 ± 145 pg/ml) nor in 12^th^ week serum TWEAK levels (responders: 704 ± 114 pg/ml vs. nonresponders: 877 ± 192 pg/ml) between the two groups (Fig. 2). Moreover neither responder (baseline: 675 ± 72 pg/ml vs 12th week: 704 ± 114 pg/ml) nor nonresponders (baseline: 547 ± 145 pg/ml vs. 12th week: 877 ± 192 pg/ml) displayed a significant modification of serum TWEAK levels between baseline and 12 weeks of etanercept administration (Fig. 2).

### Description for the first time of TWEAK-binding autoantibodies generated during PsoA

**Fig. 3:**
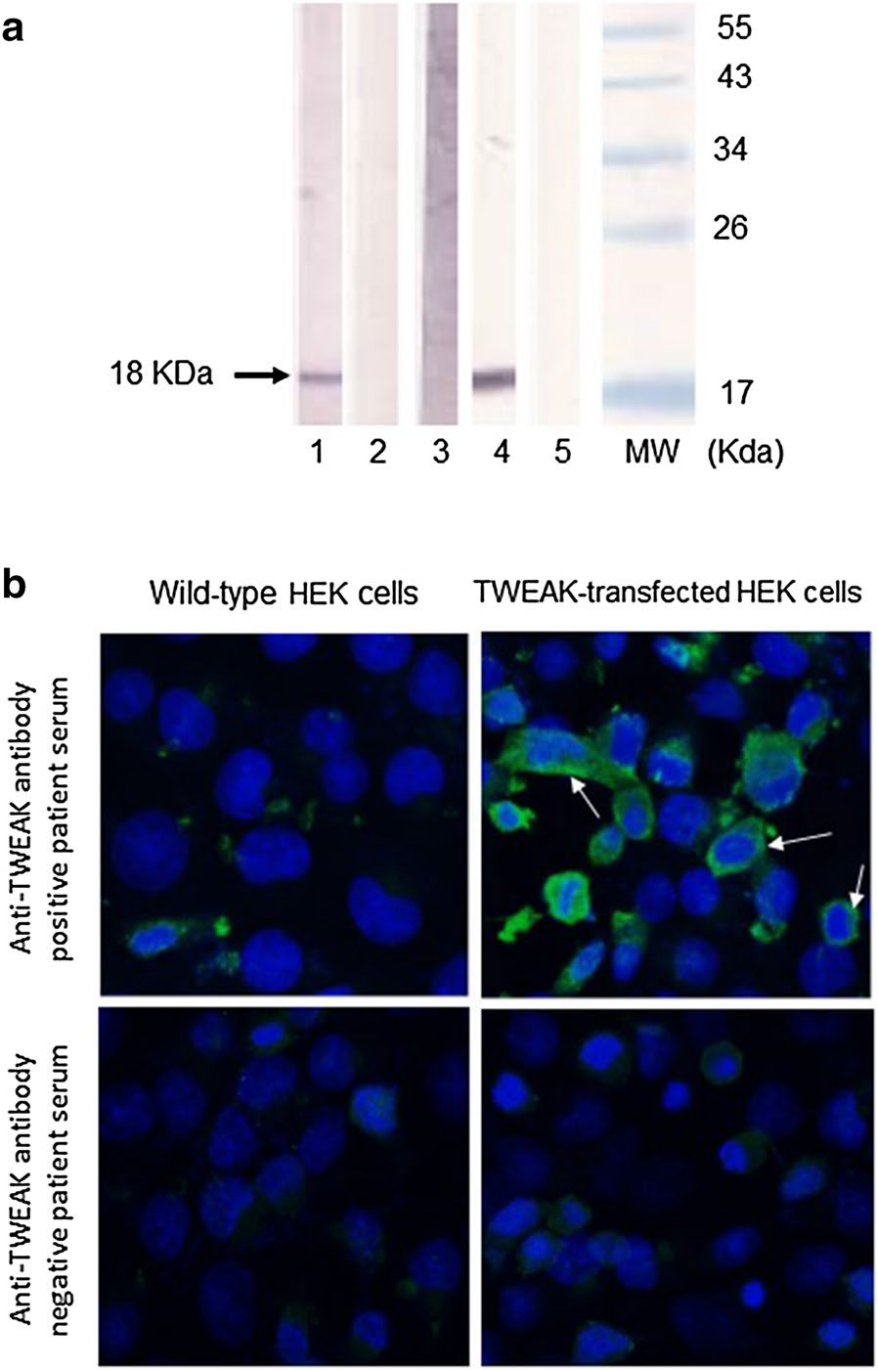
Detection of serum anti-TWEAK auto-antibodies. **a** Western blot assay using recombinant human TWEAK. *Line 1* Positive patient with PsoA. *Line 2* Negative patient with PsoA. *Line 3* Negative control serum. On line 1, 2 and 3, sera are 1:50 diluted. *Line 4* Positive control based on revelation with a commercially available polyclonal anti-TWEAK antibody. *Line 5* Negative control based on serum omission. **b** Immunofluorescence test. Serum are incubated on wild-type HEK cells and on TWEAK-transfected HEK cells. Membrane and cytoplasmic fluorescence staining patterns specifically observed with an anti-TWEAK antibody positive patient serum only on the transfected wells are indicated by *white arrows*

**Fig. 4:**
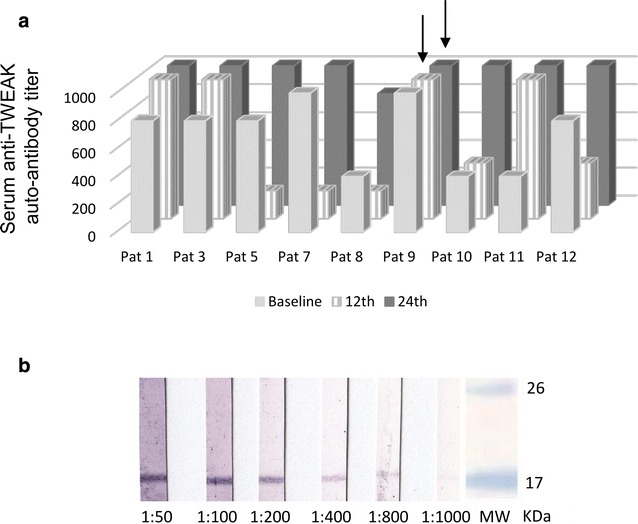
Evolution over time of serum anti-TWEAK auto-antibody titers in positive PsoA patients. **a** Titers of auto-antibodies have been obtained by serial dilutions (1:50, 1:100, 1:200, 1:400, 1:800, 1:1000) of screening positive samples (n = 9) tested with our home made western blot assay using recombinant human TWEAK. Samples with titers above 1000 are indicated by a *grey arrow*. **b** The images of each membrane according to the serial dilution are represented for a patient exhibiting TWEAK-binding IgG at a titer of 800

We were able to detect and describe for the first time anti-TWEAK autoantibodies in the serum of PsoA patients by using western blot (Fig. 3a) and/or indirect immunofluorescence (Fig. 3b). Nine out of 13 patients (corresponding to 28 samples out 39) (69.2 %) were positive for the western blot screening test while only 3 healthy controls out of 57 (5.3 %) were positive for this test (p < 0.0001). The sensitivity and the specificity of the western blot analysis for the diagnosis of PsoA were respectively 69.2 and 94.7 %. The healthy controls displayed low titers of auto-antibodies since they were all positive only at the screening dilution (1:50) while antibody titers in PsoA positive patients ranged from 200 to above 1000 (Fig. 4). Results of western blot agreed with IFI in 29/39 (74.3 %) samples and 7 samples were only positive for western blot while 3 were positive with IFI only. As shown in Fig. 4, during anti-TNF therapy, anti-TWEAK antibodies were persistent in the nine patients who were positive at baseline (Fig. 4). We did not observe the appearance of such auto-antibodies after the initiation of anti-TNF therapy in patients who were negative at baseline. In the group of the nine patients displaying anti-TWEAK antibodies in their serum, titers varied during anti-TNF therapy but the sense of variation (decrease or increase) was different from each patient to the others (Fig. 4).

### Correlation studies of serum TWEAK levels or anti-TWEAK antibody levels with clinical and biological parameters

**Fig. 5:**
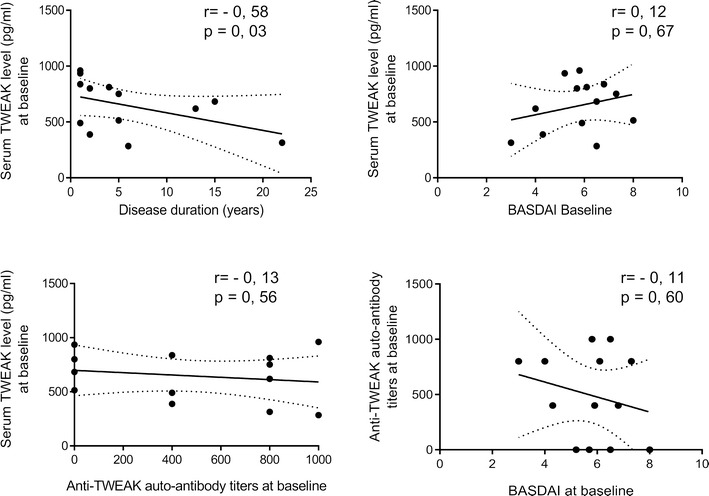
Relationship of serum TWEAK levels or anti-TWEAK antibody levels to disease duration and BASDAI. Spearman’s correlation analysis was used to test for correlations. Spearman coefficients (r) and p values (p) are indicated in each plot

In patients with PsoA, we found a significant inverse correlation (r = −0.58, p = 0.03) between serum TWEAK levels at baseline and disease duration (Fig. 5). In the same patients, we did not observe correlation between serum TWEAK levels at baseline and other parameters such as BASDAI at baseline (r = 0.12, p = 0.67), anti-TWEAK antibody titers at baseline (r = −0.13, p = 0.56) (Fig. 5) or ESR or CRP (data not shown). Anti-TWEAK antibody titers were not correlated with BASDAI neither at baseline (r = −0.11, p = 0.60) (Fig. 5) nor at 12th nor at 24th week during anti-TNF therapy (data not shown). Even if the sample size of nonresponders was too low for applying a statistical analysis, we have observed that anti-TWEAK antibody prevalence in responders (7/10 i.e. 70 %) and not responders (2/3 i.e. 66.6 %) were comparable.

### Down regulation by TWEAK-binding IgGs of the in vitro endothelial CCL-2 secretion

**Fig. 6:**
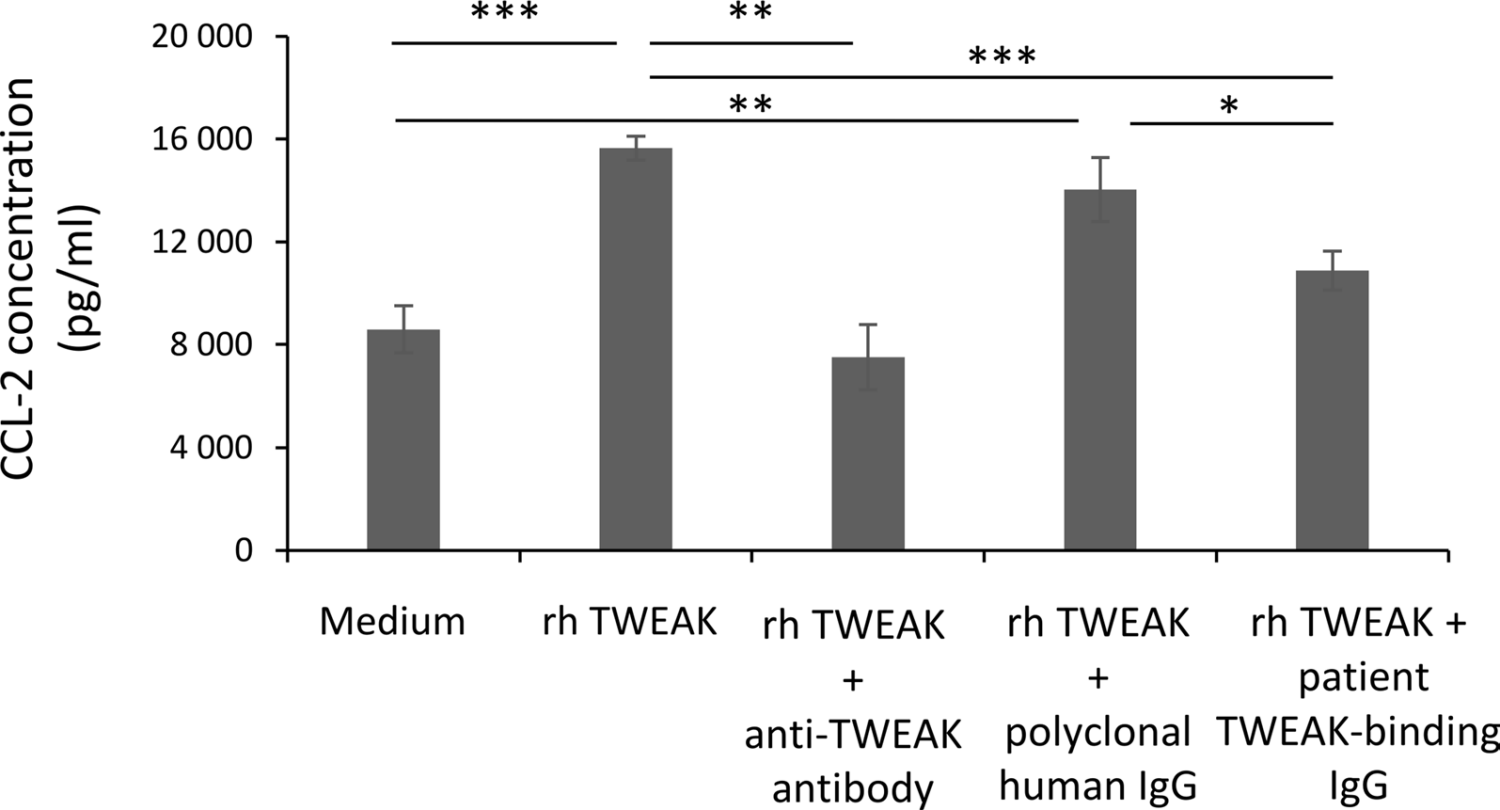
Down regulation by TWEAK-binding IgGs of the in vitro endothelium CCL-2 secretion. ELISA analysis of CCL-2 levels in the supernatants of HUVECs stimulated by TWEAK (100 ng/ml) or not (medium) and co-incubated with a commercially available goat polyclonal blocking anti-TWEAK antibody (5 µg/ml) or with purified human serum IgG negative for TWEAK-binding IgG test (50 ng/ml) or with purified PsoA patient TWEAK-binding IgG (50 ng/ml). Note the downregulation of the increased secretion of CCL-2 under TWEAK exposure when cells are incubated with purified PsoA patient TWEAK-binding IgG. *p < 0.01, **p < 0.001, ***p < 0.0001 according to Student’s t test

Inflammation is a key component of PsoA and leukocyte transmigration across the endothelium under the chemokine CCL-2 gradient is a crucial step of inflammation. To evaluate in vitro biological effects of purified PsoA patient TWEAK-binding IgG we have analyzed the impact of this antibody exposure on CCL-2 secretion by endothelial cells stimulated by rh TWEAK. We found that incubation of these cells with TWEAK-binding IgG down regulated their CCL-2 secretion under a 24 h TWEAK exposure (10 881 pg/ml with patient TWEAK-binding IgG versus 15 647 pg/ml without patient TWEAK-binding IgG; p < 0.0001) (Fig. 6).

## Discussion

Since TWEAK may be involved in PsoA pathogenesis by promoting inflammation and angiogenesis, we have investigated if beneficial effects of anti-TNF therapy in PsoA are mediated in part by blocking TWEAK produced by synovial macrophages. In the present study we have shown increased levels of serum TWEAK during PsoA at baseline. We have demonstrated that the levels of soluble TWEAK were not modulated by anti-TNF treatment and found no evidence for relationship between serum TWEAK levels and response to anti-TNF treatment. We have also described for the first time that TWEAK-binding IgG autoantibodies were generated during PsoA and were persistent at elevated titers in patients even if an etanercept therapy was conducted. In patients with PsoA, we found a significant inverse correlation between serum TWEAK levels at baseline and disease duration but not with BASDAI. Anti-TWEAK antibody titers were neither correlated with BASDAI nor with the ability to respond to anti-TNF therapy. Finally we showed that TWEAK-binding IgG down regulated CCL-2 secretion by endothelial cells stimulated by rh TWEAK.

Psoriatic arthritis (PsoA) is a heterogenous chronic inflammatory joint disease belonging to the spondyloarthropathy family that can have devastating effect on patient function and quality of life equivalent of those observed during rheumatoid arthritis (RA) [18]. TNF inhibitors have shown to work well in PsoA for axial and peripheral arthritis and the other manifestations of the disease such as cutaneous lesions of psoriasis, enthesitis and dactylitis [19]. However, given the considerable heterogeneity in the clinical manifestations in PsoA, ranging from self-limiting to chronic severe and progressive forms and concerns over safety and cost, the use of TNF blockers should be restricted to patients with an optimal risk–benefit ratio. However, validated biomarkers for predicting progression are lacking and early detection of candidates for TNF blockade remains difficult. TWEAK is a member of the TNF ligand family that functions mainly as a secreted cytokine like TNF-α [1]. TWEAK regulates a number of biological processes including cellular proliferation, angiogenesis and cytokines secretion through Fn14 [3, 20–22]. Activation of TWEAK/Fn14 signaling has been involved in several processes of injury and inflammation that result in end-organ damage including RA [23]. In 2008, Park et al. published interesting data showing that serum levels of TWEAK were significantly elevated in patients with RA, reflected disease activity and short-term response to etanercept treatment [24]. Only two publications about TWEAK involvement during PsoA are available [12, 25]. In 2009, Van Kujik et al. have found that TWEAK and its main receptor Fn14 were abundantly expressed in the inflamed synovium of RA and PsoA patients. Moreover they have demonstrated that this expression was persistent after TNF blockade by infliximab [12]. These data agreed with our results showing that another TNF blocker, etanercept, doesn’t have drastic effect on TWEAK expression. Later, Xia et al. investigated serum TWEAK levels in PsoA patients in comparizon with healthy controls [25]. They found that PsoA patients presented significantly more elevated serum TWEAK concentrations than controls. Upon concluding remarks, our work is in accordance with this study. However, we found with the same commercially available ELISA kit 10-fold higher values than Xia et al. for serum TWEAK both in patients and controls. We have recently discussed the point that the most important criticism concerning TWEAK evaluation in autoimmune/chronic inflammatory diseases is the difficulty in obtaining consensual ranges for normal values of soluble TWEAK [26]. Reagents for the assessment of TWEAK levels are now commercially available for research purposes only. However, standardization of the proposed kits is absolutely required all the more because free soluble TWEAK is a “sticky” protein difficult to manipulate based on experience from our team. We have also noted in our experience that it is very important to take particular care of pre-analytical phase to avoid TWEAK degradation in vitro and to obtain reproducible quantifications of the soluble protein. In the study of Xia, a moderate correlation was found between serum TWEAK levels and disease activity of PsoA. An explanation of the discrepancy of results of the two teams could be the use of different evaluation scores: 28-joint count Disease Activity Score (DAS 28) for Xia et al. and BASDAI for our team.

In the present study we described a new type of anti-cytokine autoantibodies occurring during chronic inflammatory disease. Anti-cytokine antibodies are described in disparate conditions and situations (cancers, infectious diseases, immunodeficiency…) and may cause a wide variety of potentially life threatening illnesses [27–29]. Anti-cytokine antibodies against TNF-α and interferon-α, two of the key players in the pathogenesis of psoriasis, have been related in psoriasis patients by Bergman et al. The authors concluded then these antibodies could be an epiphenomenon or might play a role in modulating the ongoing inflammatory process [30]. In our work, we propose that auto-immunity against another key player in the genesis of the disease, TWEAK, also take place during PsoA. The antibodies we described were of IgG isotype and circulated at high titers in the patient sera (and at low titer in controls) suggesting their belonging to auto-immune antibodies rather than to natural antibodies. We have investigated the performances of these antibodies for PsoA diagnosis and found interesting sensitivity (69.2 %) and specificity (94.7 %) but these results may be modulated by the nature of the control group we have included for the calculation of these parameters. We compared PsoA group with a healthy blood donor group but another control group with “other inflammatory diseases” was probably lacking. For detecting anti-TWEAK antibodies we developed two immunoanalysis methods, western blot and indirect immunofluorescence. Not surprisingly, we observed some discrepancies between the two approaches. Both methods were based upon a presentation of the antigen on a solid-phase but some differences in its recognition by patient antibodies can occur for following reasons: (1) antigen source in western blot was a commercial recombinant form of human TWEAK produced in *E. coli*, a protein without post-translational modifications, while TWEAK used in IFI was synthetized by human transfected cells and then post-translationally modified, (2) experimental conditions of western blot favored antigen denaturation and loss of conformational epitopes while IFI allowed conservation of native configuration of the antigen, (3) antigen expression level was relatively constant during western blot experiments while it varied with cell transfection ratio in IFI.

Our study revealed the occurrence of TWEAK-binding antibodies during PsoA. Their potential pathogenic role in PsoA remains to be examined in detail but we propose in this work that they participate in down modulating the ongoing inflammatory processes by acting on chemokine secretion, especially CCL-2, by endothelial cells and then on leukocyte chemotaxis and recruitment on the inflammatory sites. These antibodies may have a protective action in PsoA pathogenesis. However prospective in vivo studies are needed to evaluate the role of TWEAK and TWEAK-binding antibodies during PsoA.

## Conclusion

Our study revealed that during PsoA (1) serum TWEAK was up regulated and (2) TWEAK-binding autoantibodies are generated. Both parameters were not influenced by anti-TNF therapy and persisted at high levels during anti-TNF therapy. For the first time we described here TWEAK-binding IgG autoantibodies with a down regulating effect on CCL-2 secretion by endothelial cells stimulated by rh TWEAK in vitro. Finally, our results suggest that TWEAK may be involved in PsoA pathogeny.

## Abbreviations

ESR: erythrocyte sedimentation rate
FCS: fetal calf serum
HUVEC: human umbilical vein endothelial cells
PsoA: psoriatic arthritis
RA: rheumatoid arthritis
TNF: tumor necrosis factor
TWEAK: TNF weakly inducer of apoptosis

## References

[1] Chicheportiche Y, Bourdon PR, Xu H, Hsu YM, Scott H, Hession C (1997). TWEAK, a new secreted ligand in the tumor necrosis factor family that weakly induces apoptosis. J Biol Chem.

[2] Desplat-Jégo S, Varriale S, Creidy R, Terra R, Bernard D, Khrestchatisky M (2002). TWEAK is expressed by glial cells, induces astrocyte proliferation and increases EAE severity. J Neuroimmunol.

[3] Desplat-Jégo S, Creidy R, Varriale S, Allaire N, Luo Y, Bernard D (2005). Anti-TWEAK monoclonal antibodies reduce immune cell infiltration in the central nervous system and severity of experimental autoimmune encephalomyelitis. Clin Immunol.

[4] Desplat-Jego S, Feuillet L, Creidy R, Malikova I, Rance R, Khrestchatisky M (2009). TWEAK is expressed at the cell surface of monocytes during multiple sclerosis. J Leukoc Biol.

[5] Winkles JA (2008). The TWEAK-Fn14 cytokine-receptor axis: discovery, biology and therapeutic targeting. Nat Rev Drug Discov.

[6] Zhao Z, Burkly LC, Campbell S, Schwartz N, Molano A, Choudhury A (2007). TWEAK/Fn14 interactions are instrumental in the pathogenesis of nephritis in the chronic graft-versus-host model of systemic lupus erythematosus. J Immunol.

[7] Campbell S, Michaelson J, Burkly L, Putterman C (2004). The role of TWEAK/Fn14 in the pathogenesis of inflammation and systemic autoimmunity. Front Biosci.

[8] Perper SJ, Browning B, Burkly LC, Weng S, Gao C, Giza K (2006). TWEAK is a novel arthritogenic mediator. J Immunol.

[9] Zheng TS, Burkly LC (2008). No end in site: TWEAK/Fn14 activation and autoimmunity associated-end-organ pathologies. J Leukoc Biol.

[10] Kamata K, Kamijo S, Nakajima A, Koyanagi A, Kurosawa H, Yagita H (2006). Involvement of TNF-like weak inducer of apoptosis in the pathogenesis of collagen-induced arthritis. J Immunol.

[11] Kamijo S, Nakajima A, Kamata K, Kurosawa H, Yagita H, Okumura K (2008). Involvement of TWEAK/Fn14 interaction in the synovial inflammation of RA. Rheumatology (Oxford).

[12] van Kuijk AW, Wijbrandts CA, Vinkenoog M, Zheng TS, Reedquist KA, Tak PP (2010). TWEAK and its receptor Fn14 in the synovium of patients with rheumatoid arthritis compared to psoriatic arthritis and its response to tumour necrosis factor blockade. Ann Rheum Dis.

[13] Coates LC, Kavanaugh A, Ritchlin CT (2014). Systematic review of treatments for psoriatic arthritis: 2014 update for the GRAPPA. J Rheumatol.

[14] Gladman DD, Antoni C, Mease P, Clegg DO, Nash P (2005). Psoriatic arthritis: epidemiology, clinical features, course, and outcome. Ann Rheum Dis.

[15] Taylor WJ, Harrison AA (2004). Could the Bath Ankylosing Spondylitis Disease Activity Index (BASDAI) be a valid measure of disease activity in patients with psoriatic arthritis?. Arthritis Rheum.

[16] Wendling D, Lukas C, Paccou J, Claudepierre P, Carton L, Combe B (2014). Recommendations of the French Society for Rheumatology (SFR) on the everyday management of patients with spondyloarthritis. Joint Bone Spine.

[17] Garrett S, Jenkinson T, Kennedy LG, Whitelock H, Gaisford P, Calin A (1994). A new approach to defining disease status in ankylosing spondylitis: the Bath Ankylosing Spondylitis Disease Activity Index. J Rheumatol.

[18] Sokoll KB, Helliwell PS (2001). Comparison of disability and quality of life in rheumatoid and psoriatic arthritis. J Rheumatol.

[19] Kavanaugh AF, Ritchlin CT (2006). Systematic review of treatments for psoriatic arthritis: an evidence based approach and basis for treatment guidelines. J Rheumatol.

[20] Chicheportiche Y, Chicheportiche R, Sizing I, Thompson J, Benjamin CB, Ambrose C (2002). Proinflammatory activity of TWEAK on human dermal fibroblasts and synoviocytes: blocking and enhancing effects of anti-TWEAK monoclonal antibodies. Arthritis Res.

[21] Lynch CN, Wang YC, Lund JK, Chen YW, Leal JA, Wiley SR (1999). TWEAK induces angiogenesis and proliferation of endothelial cells. J Biol Chem.

[22] Wiley SR, Winkles JA (2003). TWEAK, a member of the TNF superfamily, is a multifunctional cytokine that binds the TweakR/Fn14 receptor. Cytokine Growth Factor Rev.

[23] Burkly LC, Michaelson JS, Hahm K, Jakubowski A, Zheng TS (2007). TWEAKing tissue remodeling by a multifunctional cytokine: role of TWEAK/Fn14 pathway in health and disease. Cytokine.

[24] Park MC, Chung SJ, Park YB, Lee SK (2008). Relationship of serum TWEAK level to cytokine level, disease activity, and response to anti-TNF treatment in patients with rheumatoid arthritis. Scand J Rheumatol.

[25] Xia L, Shen H, Xiao W, Lu J (2011). Increased serum TWEAK levels in Psoriatic arthritis: relationship with disease activity and matrix metalloproteinase-3 serum levels. Cytokine.

[26] Bertin D, Stephan D, Khrestchatisky M, Desplat-Jego S (2013). Is TWEAK a biomarker for autoimmune/chronic inflammatory diseases?. Front Immunol.

[27] Browne SK, Burbelo PD, Chetchotisakd P, Suputtamongkol Y, Kiertiburanakul S, Shaw PA (2012). Adult-onset immunodeficiency in Thailand and Taiwan. N Engl J Med.

[28] Browne SK, Holland SM (2010). Anticytokine autoantibodies in infectious diseases: pathogenesis and mechanisms. Lancet Infect Dis.

[29] Burbelo PD, Browne SK, Sampaio EP, Giaccone G, Zaman R, Kristosturyan E (2010). Anti-cytokine autoantibodies are associated with opportunistic infection in patients with thymic neoplasia. Blood.

[30] Bergman R, Ramon M, Wildbaum G, Avitan-Hersh E, Mayer E, Shemer A (2009). Psoriasis patients generate increased serum levels of autoantibodies to tumor necrosis factor-alpha and interferon-alpha. J Dermatol Sci.

